# Ray–Wave Correspondence in Anisotropic Mesoscopic Billiards

**DOI:** 10.3390/e27020132

**Published:** 2025-01-26

**Authors:** Martina Hentschel, Samuel Schlötzer, Lukas Seemann

**Affiliations:** Institute of Physics, Technische Universität Chemnitz, D-09107 Chemnitz, Germany; samuel.schloetzer@s2021.tu-chemnitz.de (S.S.); lukas.seemann@physik.tu-chemnitz.de (L.S.)

**Keywords:** mesoscopic billiards, anisotropy, birefringence, ray–wave correspondence, phase-space dynamics, optical microcavities, bilayer graphene, index ellipsoid, dispersion relation

## Abstract

Mesoscopic billiard systems for electrons and light, realized as quantum dots or optical microcavities, have enriched the fields of quantum chaos and nonlinear dynamics not only by enlarging the class of model systems, but also by providing access to their experimental realization. Here, we add yet another system class, two-dimensional billiards with anisotropies. One example is the anisotropic dispersion relation relevant in bilayer graphene known as trigonal warping, and another is the birefringent closed optical disk cavity. We demonstrate that the established concept of ray–wave correspondence also provides useful insight for anisotropic billiard systems. First, we approach the dynamics of the anisotropic disk from the ray-tracing side that takes the anisotropy in momentum space into account, based on the non-spherical index ellipsoid. Second, we use transformation optics to solve the wave problem and find the resonances to be those of the isotropic elliptical cavity. We illustrate ray–wave correspondence and mark differences in the description of optical and electronic anisotropic systems.

## 1. Introduction

Symmetries and their breaking have been an inspiring principle throughout physics, with the development of the standard model of particle physics and the gauge symmetries used to derive quantum field theories as prominent examples [1]. They rely on the Noether theorem [2], in which Emmy Noether related continuous symmetries to conserved quantities—and vice versa—more than 100 years ago. Furthermore, the ongoing progress in material sciences and mesoscopic physics [3] constantly introduces novel physical systems with various properties to explore and manipulate. The fabrication of two-dimensional electron gas enabled the investigation of ballistic electronic quantum dot billiards and boosted the field of quantum chaos [4,5], as did optical microcavities [6,7]. The variety of these systems increases the pool of properties that can be studied. For example, bilayer graphene (BLG) systems can provide an anisotropic dispersion relation in momentum space [8,9], which was found to tremendously affect the internal billiards dynamics, even for circular confinement [10]. Magnetic anisotropies play a role in the dynamics of nanomagnets subject to magnetoelectric interaction [11,12] and straintronics [13]. Anisotropic optical systems and metamaterials are receiving increasing interest [14,15,16,17,18].

The theoretical description of smaller and smaller billiard systems calls for their quantum description, that remains a challenge for mesoscopic systems with typical sizes on the micrometer scale—several orders of magnitude larger than typical quantum systems, yet corresponding to system sizes of only several wavelengths across. Semiclassical methods [4,5,19] are useful and provide deeper insight, with the concept of classical–quantum, or ray–wave, correspondence being particularly straightforward to apply. This fundamental principle provides a qualitative understanding of the dynamics, especially in ballistic mesoscopic billiards [20,21,22,23,24], based on the description of their classical counterparts. Moreover, it allows for a (semi)quantitative prediction of their emission properties not only for optical microresonators [25], but also for electronic systems like single-layer graphene [26].

How does a loss of symmetry in a system caused by the presence of anisotropies change its dynamics? For instance, let us consider whispering gallery (WG) orbits that directly reflect the isotropy of space and, according to Noether’s theorem, the conservation of angular momentum in rotational invariant cavities. How do these trajectories change in the presence of anisotropies, i.e., in the presence of *preferred* propagation directions that break the rotational invariance of the system.

Here, we want to explore the chances of a semiclassical description of anisotropic mesoscopic billiards, with BLG billiards and the birefringent optical disk as examples, for an electronic and an optical system, respectively. We will first briefly review the particle tracing in BLG systems [10] and outline its generalization to anisotropic optical systems. While a wave description of condensed-matter systems typically requires tight-binding modeling, Maxwell’s equation allows for a description of anisotropic dielectric systems using transformation optics (TO). We illustrate TO for the birefringent disk cavity and are able to establish the expected ray–wave correspondence. However, this does not hold for the BLG case where the presence of two so-called valleys associated with the presence of two (non-identical) Dirac points changes the situation. We end this paper with a summary and a future outlook for research in this field.

## 2. Trajectory Tracing in Electronic and Optical Anisotropic Billiards

The description of the particle dynamics in generic billiards is a well-established field of nonlinear dynamics [27] and is well understood both in real space—showing the trajectories—and phase space, where, typically, a Poincaré surface of section (PSOS) is taken at the billiard’s boundary to reduce the number of phase-space variables from four (as we consider two-dimensional systems) to two, namely the boundary coordinate *s* and sinχ (the sine of the angle of incidence). In isotropic circular billiards, the PSOS consists of points along a line of constant sinχ, directly expressing the conserved angular momentum.

### 2.1. Anisotropic Bilayer Graphene Billiards

**Figure 1:**
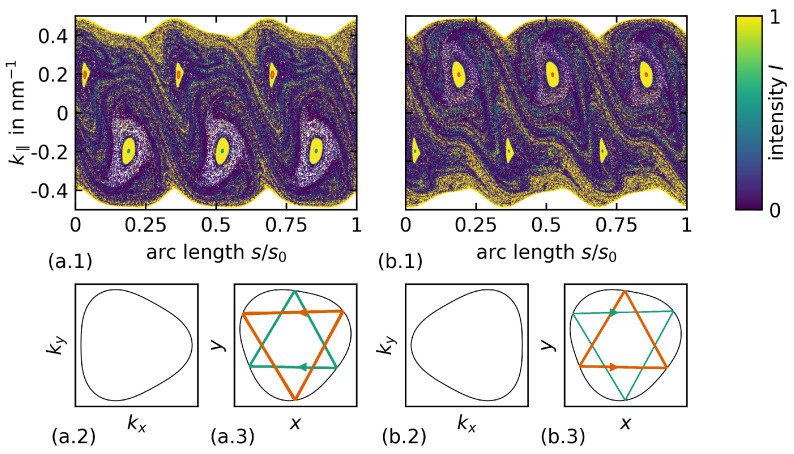
The Poincaré surface of section (PSOS) for a bilayer graphene (BLG) billiard of onigiri shape with $\varepsilon_{3}=0.08$ and a tilt angle of $\vartheta_{0}=20^{\circ }$ between the BLG lattice and geometry symmetry axis weighted by the trajectory intensity (color scale). (**a.1**) The PSOS for the $K^{+}$ valley; (**b.1**) the PSOS for the $K^{-}$ valley. The islands correspond to the triangular orbits indicated in panels (**a.3**,**b.3**) and are a consequence of the anisotropic dispersion relation (or Fermi line) in momentum space shown in (**a.2**,**b.2**). The difference in the PSOS is directly related to the different (mirror-symmetry-related) Fermi lines in (**a.2**,**b.2**); however, each PSOS reflects an anisotropic situation. The position of the triangular orbits results from the interplay of preferred propagation directions perpendicular to the flat sides of the Fermi line and cavity-geometry-supported stable trajectories [28]. The high intensity (yellow color) at the borders of the PSOS indicate whispering gallery (WG)-type trajectories that evolve towards the chaotic sea. The initial conditions were chosen slightly differently in (**a.1**,**b.1**).

The bilayer of graphene billiards has to change in the presence of anisotropies, even when the circular billiard geometry is kept: when space is anisotropic, Noether’s theorem [2] states that angular momentum cannot be conserved any more. For BLG, such an anisotropy results from its band structure (obtained from a 4×4 tight-binding Hamiltonian), which is found to not be rotationally invariant around its band minima and maxima (the so-called *K* points) for certain yet generic parameter choices. The intersection curve of the band at a certain energy (the Fermi energy) defines the dispersion relation or Fermi line, which then differs from a circle and is thus anisotropic. Its shape is known as trigonal warping, a deformed circle where three corner-like features emerge (see Figure 1(a.2,b.2)), and is related by mirror symmetry at the two *K* points (or valleys), $K^{+}$ and $K^{-}$, that result from pseudospin (the presence of two atoms per unit cell). For more details on graphene physics, we would like to refer the interested reader to Refs. [8,9,10], and leave others with the information that trigonal warping of the energy bands in BLG is an expression of anisotropy.

The PSOS for such an anisotropic BLG billiard is shown in Figure 1. This example shows the PSOS for a BLG cavity with a onigiri geometry, which somewhat resembles the shape of the BLG Fermi line and is given in polar coordinates as $r\left(\varphi \right)=R_{0}(1+\varepsilon_{3}\cos\left(3\left(\varphi -\vartheta_{0}\right)\right)$). Here, $\varepsilon_{3}$ is the geometric shape parameter and $\vartheta_{0}$ gives the angle of rotation between the underlying BLG lattice and the billiard’s axial symmetry axis.

First of all, we have to notice that the PSOS is not spanned by *s* and sinχ, but rather, by *s* and $k_{\|}$ (with $k_{\|}$ > 0 indicating a mathematically positive sense of rotation): as the angle of incidence cannot be equal to the angle of reflection in general anymore, we have to find another quantity that is conserved upon reflection at the boundary. Indeed, the momentum component parallel to the interface is conserved upon reflection and is therefore a suitable phase space variable. The curly upper and lower edges of the PSOS are caused by the non-circular shape of the Fermi line. The maximum possible value of the $k_{\|}$ component changes depending on the orientation of the billiard boundary to the Fermi line (which is fixed to the BLG lattice). It is striking to see in Figure 1(a.1,b.2) that the PSOS is not symmetric with respect to $k_{\|}=0$ anymore (for a single *K* point), in contrast to what applies for isotropic billiards. At this point, we have to note that the PSOS corresponding to the two K points (with Fermi lines related by mirror symmetry) are not identical (Figure 1(a.1,b.1)). Notice that symmetry with respect to $k_{\|}$ = 0 is restored when both *K* points are considered.

The PSOS in Figure 1 contains intensity information (the reflection coefficient is derived from the BLG Hamilton operator and taken into account at each reflection [10]), with light colors indicating high intensity. It is striking that there are two chains of three islands that correspond to triangular orbits built from the three preferred propagation directions present in a trigonally warped Fermi line. Their shapes illustrate that the Fermi lines and PSOS corresponding to the two different *K* points are related by mirror symmetry. In turn, WG orbits are lost as they turn into chaotic trajectories.

### 2.2. Anisotropy and Polarization

We now turn to optical systems that are also governed by wave equations. An optical or electromagnetic wave with frequency ω is described by Maxwell’s equations. It is given by its wave vector $\vec{k}$, the electric field $\vec{E}$, the electric displacement field $\vec{D}=\varepsilon_{0}\hat{\varepsilon }\vec{E}$, the magnetic field strength $\vec{H}$, and the magnetic induction $\vec{B}=\mu_{0}\hat{\mu }H$ with the vacuum permittivity (permeability) $\varepsilon_{0}$ ($\mu_{0}$), the permittivity tensor $\hat{\varepsilon }$, and the permeability tensor $\hat{\mu }$. The Poynting vector $\vec{S}=\left(\vec{E}\times \vec{H}*\right)/2$ describes the flow direction of the energy flux. The Maxwell’s equations in anisotropic materials that we are interested in here connect the following quantities:

(1) $$\vec{\nabla }\times \vec{E}=-\dot{\vec{B}}=i\omega \mu_{0}\hat{\mu }\vec{H}\;,$$

(2) $$\vec{\nabla }\times \vec{H}=\dot{\vec{D}}=-i\omega \varepsilon_{0}\hat{\varepsilon }\vec{E}\;,$$

and we will use them whenever we argue in terms of the wave picture.

**Figure 2:**
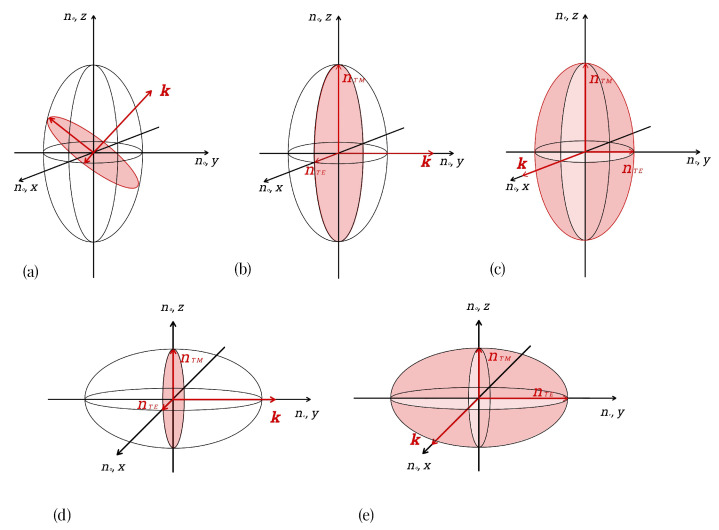
An index ellipsoid for a uniaxial optical system with refractive indices $n_{o}$ and $n_{e}$. (**a**) shows the generic case. The wave vector $\vec{k}$ always lies in the resonator plane. The intersectional plane perpendicular to $\vec{k}$ defines the relevant refractive indices as the semi-axis of the intersectional ellipse (marked by red arrows). In (**b**,**c**), the extraordinary refractive index is pointing out of the resonator plane. The intersection plane perpendicular to $\vec{k}$ through the ellipsoid center has the same shape independent of the direction of $\vec{k}$. In particular, the refractive index for TM (TE) does not vary with $\vec{k}$ and remains constant at $n_{\mathrm{TM}}=n_{e}$ ($n_{\mathrm{TE}}=n_{o}$). In (**d**,**e**), the optical axis lies in the resonator plane. Now, the intersectional plane changes its shape as $\vec{k}$ varies. However, its shape varies such that the minor semi-axis remains at a constant value $n_{o}$. Since it is always perpendicular to the resonator plane, we identify it as the refractive index relevant for TM polarization, $n_{\mathrm{TM}}=n_{o}$, instead of $n_{e}$ as in (**b**,**c**). This polarization behaves as usual and corresponds to the ordinary ray. The other polarization, TE, experiences a refractive index that varies with the direction of $\vec{k}$. This induces birefringent behavior and is the case we are mainly studying here.

For now, we want to generalize the ray-tracing algorithm developed for BLG billiards [10] to anisotropic optical systems. To this end we have to use insight from the wave picture as an ingredient for the ray description, very similar to what we discussed for BLG. We consider a uniaxial crystal such as calcite where the non-cubic lattice or a layered structure is the origin of an anisotropy known as birefringence. We assume a refractive index $n_{e}$ in one direction, and indices $n_{o}$ along the remaining two Cartesian coordinates. An example of the resulting index ellipsoid is shown in Figure 2a for a generic wave vector $\vec{k}$. According to Maxwell’s equations, the $\vec{D}$ field oscillates in a plane perpendicular to $\vec{k}$, indicated in red. The magnetic field $\vec{H}$ is perpendicular to the plane spanned by $\vec{D}$ and $\vec{k}$ [29,30]. The boundary of the intersectional plane, which results from the intersection between the $\vec{D}$-$\vec{H}$ plane and the ellipsoid, forms an ellipse, and its two semi-axes define the refractive indices for the two polarizations.

To explain the interplay between anisotropy in a two-dimensional optical system and the polarization state of light, let us consider a simpler case where the resonator plane lies in the (x,y) plane. We then refer to polarization where the electric (magnetic) field $E_{z}$ ($H_{z}$) oscillates in the *z* direction as TM (TE) polarization while the magnetic (electric) field is transverse to the *z* axis and lies in the resonator plane. If now, the lattice direction with the (larger) refractive index $n_{e}$ is aligned along the *z* axis (Figure 2b,c), it follows from Maxwell’s equations that the TM modes behave like those of an isotropic disk with refractive index $n_{e}$. The TE modes are those with $n_{o}$ and thus remain unchanged compared to the isotropic case; they are referred to as ordinary rays. For these, the magnetic field oscillates in the resonator plane and does not experience any anisotropy. This is in agreement with the argumentation based on the index ellipsoid illustrated in Figure 2b,c. Here, all intersectional planes perpendicular to $\vec{k}$ and through the center of the ellipsoid possess the same elliptical cross section. For TM polarization, the electric field $\vec{E}$ is aligned with the *z* axis and experiences a refractive index equal to $n_{e}$, the semi-axis in the *z* direction of the intersectional ellipse. In contrast, for TE polarization, the electric field $\vec{E}$ lies in the (x,y) plane, where it will experience the refractive index $n_{o}$ for any $\vec{k}$.

The more interesting case is the one where the larger refractive index $n_{e}$ is, without loss of generality, pointing in the *y* direction (Figure 2d,e). Again, there are no changes for TM polarization that behaves as in the isotropic case, but now corresponding to a refractive index $n_{o}$, where the anisotropy in the resonator (x,y) plane is not accessible to an electric field oscillating perpendicular to it. In the line of reasoning based on the index ellipsoid, we argue that the intersectional plane will always possess a semi-axis of size $n_{o}$ in the *z* direction. The one case remaining, a TE polarization when $n_{e}$ lies in the resonator plane, is the most interesting one, with qualitatively new behavior that differs from the isotropic disk. We will focus on this case in the following section.

### 2.3. From the Index Ellipsoid to the Dispersion Relation in Uniaxial Optical Billiards

The dispersion relation, i.e., the relation between the frequency ω of light and its wave vector $\vec{k}$, that we need to generalize the trajectory tracing algorithm developed for BLG billiards to the optical case can be directly deduced from the index ellipsoid. We recall the generic equation for an ellipsoid in (x,y,z) space with semi-axis $b_{1},b_{2}$ and $b_{3}$ to be $x^{2}/b_{1}^{2}+y^{2}/b_{2}^{2}+z^{2}/b_{3}^{2}=1$. If the refractive index in the *y* direction is largest, we thus obtain an index ellipsoid elongated in the *y* direction in real space.

**Figure 3:**
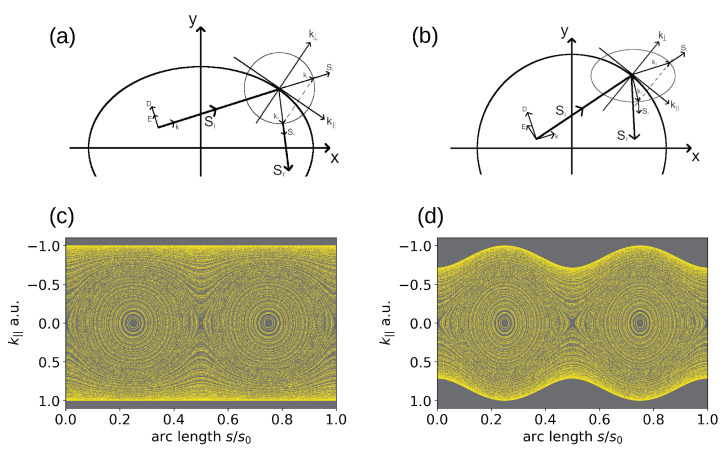
A single reflection as a basic building block of ray trajectory tracing in (**a**) an isotropic elliptical billiard and (**b**) an anisotropic disk billiard. The thick (thin) black line indicates the cavity geometry (dispersion relation). While the angles of incidence and reflection are equal in the isotropic case (**b**), this is not the case in the anisotropic situation (**a**). The resulting PSOS is shown in (**c**) for the closed isotropic ellipse and (**d**) for the anisotropic disk with an elliptic dispersion relation. Both ellipses, in real and momentum space in (**c**) and (**d**), respectively, have the same eccentricity corresponding to a=0.7.

While $\vec{E}$ and $\vec{D}$; $\vec{H}$ and $\vec{B}$; and $\vec{S}$ and $\vec{k}$, respectively, point in the same direction in an isotropic material (Figure 3a), this is not the case in anisotropic media (cf. Figure 3b). The isotropic condition $\vec{E}⊥\vec{B}⊥\vec{k}$ is replaced by $\vec{D}⊥\vec{k}$ and $\vec{S}⊥\vec{E}$. The vectors $\vec{D}$, $\vec{E},\vec{k}$, and $\vec{S}$ are all in one plane, and $\vec{H}$ and $\vec{B}$ are perpendicular to it. We refer the reader to Ref. [29] for details. Maxwell’s equations yield the relation

(3) $$\vec{k}\times \left(\vec{k}\times \vec{E}\right)=-\omega^{2}\mu_{0}\hat{\varepsilon }\vec{E}\;.$$

providing the desired relation between ω and $\vec{k}$, the dispersion relation [31], in the form of an eigenvalue problem for $\vec{E}$. The existence of non-trivial solutions requires the determinant yielding the characteristic polynomial to vanish. The relation derived from this condition is referred to as the dispersion relation [29].

In the special case of a uniaxial crystal with $n_{e}>n_{o}$ in, say, the *y* direction that we consider here, the situation simplifies as the expression for the determinant factorizes. As each factor can become zero, we find two solutions: one dispersion relation is spherical (and involves only $n_{o}$) and represents the ordinary wave, while the other possibility is a spheroid associated with the extraordinary wave we are particularly interested in here. It corresponds to a dispersion ellipse that is elongated in the $k_{x}$ direction in momentum space, with the length of the semi-axes being directly related to the refractive indices such that the semi-axis in the $k_{x}$ direction is proportional to $n_{e}$, while the one in the $k_{y}$ direction is governed by $n_{o}$. Thus, the dispersion directly reflects the anisotropy of the system, and we introduce the anisotropy parameter *a*, 0≤a<1,

(4) $$a=\sqrt{1-\frac{n_{o}^{2}}{n_{e}^{2}}}\;.$$

We add a qualitative argument to make the rotation of the ellipse (from an index ellipsoid elongated in the *y* direction to a dispersion relation elongated in the $k_{x}$ direction) required when going from the index ellipsoid to momentum space more plausible. In a medium with refractive index *n*, the wavelength (a measure in real space x,y,z) decreases by *n* in comparison to a vacuum, while the wave number (related to momentum space ($k_{x},k_{y},k_{z}$) increases by *n*. This reciprocal relation between real and momentum space (we mention a similar reciprocity when constructing the reciprocal lattice, where large reciprocal lattice vectors correspond to small distances between the associated lattice planes and vice versa) suggests a dispersion relation elongated in the $k_{x}$ direction with the equation of an ellipse

(5) $$k_{x}^{2}+\frac{n_{e}^{2}}{n_{o}^{2}}k_{y}^{2}=k_{x}^{2}+\frac{k_{y}^{2}}{1-a^{2}}=1,$$

with the anisotropy parameter *a* defined in Equation (4). Its consequences for the reflection at the wall of an anisotropic billiard are illustrated in Figure 3b) in comparison to the inverted isotropic situation (circular dispersion relation, now with an elliptical real-space geometry) in Figure 3a).

**Figure 4:**
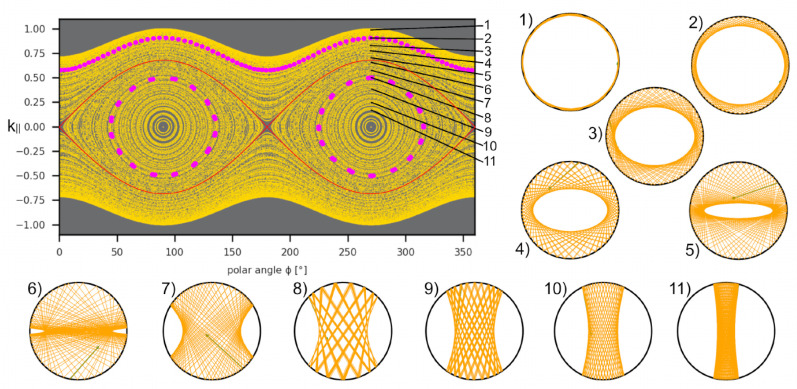
The PSOS of the anisotropic closed disk with the anisotropy parameter a=0.7. Although we consider a circular cavity, the phase space clearly resembles that of a closed ellipse. The insets (1) to (11) show sample trajectories. Examples (1) to (5) correspond to WG-type trajectories, while (7) to (11) show bouncing-ball orbits. Trajectory (6) is very close to the separatrix and illustrates how the switch between the two kinds of trajectories occurs. The small green arrows visible in (4) to (7) indicate the ray’s starting condition.

The PSOSs resulting from ray tracing for the situations depicted in Figure 3a,b, i.e., for a closed isotropic ellipse and an anisotropic disk, both with the same eccentricity parameter corresponding to a=0.7, are shown in Figure 3c,d for comparison. They resemble each other [32,33], up to the curly boundary limits arising from the non-circular dispersion relation, as discussed above. The striking characteristics of the PSOS of the anisotropic disk billiards (cf. also Figure 4) are two islands around central elliptic fixed points, in analogy to the PSOS of the isotropic elliptic billiards, where they represent so-called bouncing-ball orbits that go back and forth between the flat sides of the ellipse. In our anisotropic case, the bouncing-ball trajectories illustrated in Figure 4(7–11) arise from the existence of preferred propagation directions in momentum space, corresponding to flat sides of the ellipse of (now) the dispersion relation. The group velocities in these regions, given by the normal to the dispersion relation, combine to form bouncing-ball orbits (similar to the triangular orbits seen in BLG billiards above (cf. also Figure 3)) along the diameter. In the case of a circular dispersion relation, diameter orbits exist in all directions but are not stable (rather, they are marginally stable). For the anisotropic case, there is also a pair of unstable fixed points in between the stable (bouncing-ball) ones, indicating a diameter orbit formed by the group velocities at the points of highest curvature of the elliptic dispersion relation.

In addition, there are WG-type trajectories that do not change the sense of their propagation direction and travel along the billiard boundary (cf. Figure 4(1–5)). Their trace in phase space depends on their sense of rotation and lies outside the island region towards the upper (lower) PSOS edge for counterclockwise (clockwise) propagation. These regions (of WG and bouncing-ball orbits, respectively) are disjoined by the separatrix, and the trajectory in cf. Figure 4(6) shows a sample trajectory very close to the separatrix (but slightly on the bouncing-ball side).

**Figure 5:**
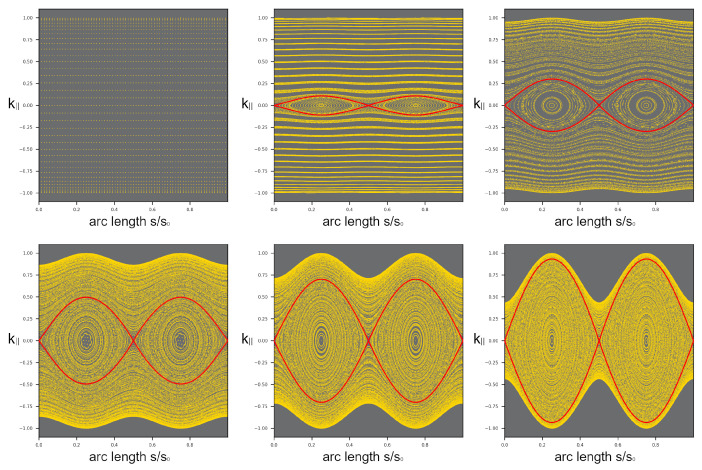
The PSOS for the closed anisotropic disk, visualizing $k_{\|}$ (normalized by its maximum) over the arc length $s/s_{0}$ for anisotropy parameters *a* = 0, 0.1, 0.3, 0.5, 0.7, 0.9 (from top left to bottom right). The red lines represent separatrixes which distinguish between bouncing-ball and WG-type trajectories.

The evolution of the PSOS of the anisotropic disk with an increasing anisotropy parameter *a* is shown in Figure 5. Starting from the isotropic disk, a=0, with a PSOS consisting of WG orbits represented by straight lines, the bouncing-ball island signature becomes the dominating structure in phase space as *a* increases. Larger *a* correspond to higher anisotropy and the stronger formation of preferred propagation directions. This is consistent with a larger portion of bouncing-ball orbits, as these are composed of these preferred propagation directions. The red line denotes the separatrix that separates these two regions of qualitatively different trajectory dynamics.

## 3. Wave and Transformation Optics for the Birefringent Disk

We start our considerations with Maxwell’s equations, which we have to solve for the electric field $\vec{E}$ and the magnetic field $\vec{H}$ for an anisotropic medium with permittivity tensor $\hat{\varepsilon }$ and permeability tensor $\hat{\mu }$.

(6) $$\vec{\nabla }\times {\hat{\mu }}^{-1}\left(\vec{\nabla }\times \vec{E}\right)=\frac{\omega^{2}}{c^{2}}\hat{\varepsilon }\vec{E}$$

(7) $$\vec{\nabla }\times {\hat{\varepsilon }}^{-1}\left(\vec{\nabla }\times \vec{H}\right)=\frac{\omega^{2}}{c^{2}}\hat{\mu }\vec{H}\;\;\;.$$

While their solution is straightforward for some specific anisotropic solutions, as argued above, when discussing this in terms of the index ellipsoid, their generic solution states a mathematical challenge [34,35]. In physics, transformation optics provides a well-established method to overcome these mathematical problems.

The central idea of transformation optics [16,36] is the constancy of the optical path length, which is the product of the refractive index times the geometric path length. If there is, for example, an anisotropy in the refractive index in one direction, one may rescale this refractive index by ’counterscaling’ the respective spatial direction (such that it fits the other ones and, thus, yields an isotropic permittivity tensor $\hat{\varepsilon }$). It is essential to note that stretching or compressing the coordinate system in a given direction modifies the permeability tensor $\hat{\mu }$ even though its components are uniform in the original system.

Here, we assume an anisotropy in the *y* direction and focus on TE polarized light, as before. The corresponding coordinate transformation from the old coordinates x,y,z to the new ones u,v,z thus reads (cf. [18])

(8) (u,v,z)=(x,βy,z)

with

(9) $$\beta =\frac{n_{o}}{n_{e}}\;.$$

While requiring constancy of the optical path length readily yields the stretching factor β, it can also be derived via the permittivity tensor. Our starting point is the anisotropic situation

(10) $$\hat{\varepsilon }=\left(\begin{array}{ccc} n_{o}^{2} & 0 & 0\\ 0 & n_{e}^{2} & 0\\ 0 & 0 & n_{o}^{2} \end{array}\right)\;,\;\;\;\;\;\hat{\mu }=\left(\begin{array}{ccc} 1 & 0 & 0\\ 0 & 1 & 0\\ 0 & 0 & 1 \end{array}\right)$$

and the following ansatz for the transformation matrix $\hat{A}$:

(11) $$\hat{A}=\left(\begin{array}{ccc} 1 & 0 & 0\\ 0 & \beta & 0\\ 0 & 0 & 1 \end{array}\right)\;.$$

The transformed quantities (denoted by ′) in the transformed coordinates (u,v,z) follow from $\hat{A}$ as ${\vec{a}}^{\prime }=\hat{A}\vec{a}$ for vectors, while matrices fulfill the relation [18]

(12) $${\hat{M}}^{\prime }=\frac{1}{\det\hat{A}}\hat{A}\hat{M}{\hat{A}}^{T}\;.$$

We thus obtain

(13) $${\hat{\varepsilon }}^{\prime }=\left(\begin{array}{ccc} \frac{1}{\beta } & 0 & 0\\ 0 & \beta & 0\\ 0 & 0 & \frac{1}{\beta } \end{array}\right)\hat{\varepsilon }=\left(\begin{array}{ccc} \frac{n_{o}^{2}}{\beta } & 0 & 0\\ 0 & \beta n_{e}^{2} & 0\\ 0 & 0 & \frac{n_{o}^{2}}{\beta } \end{array}\right)\;,\;\;\;\;\;{\hat{\mu }}^{\prime }=\left(\begin{array}{ccc} \frac{1}{\beta } & 0 & 0\\ 0 & \beta & 0\\ 0 & 0 & \frac{1}{\beta } \end{array}\right)\hat{\mu }=\left(\begin{array}{ccc} \frac{1}{\beta } & 0 & 0\\ 0 & \beta & 0\\ 0 & 0 & \frac{1}{\beta } \end{array}\right)\;,$$

yielding a transformed isotropic permittivity tensor ${\hat{\varepsilon }}^{\prime }$ when choosing $\beta =n_{o}/n_{e}$. Note that ${\hat{\mu }}^{\prime }$ is now anisotropic such that ${\hat{\varepsilon }}^{\prime }$ and ${\hat{\mu }}^{\prime }$ have changed their roles under the transformation. The dispersion relation remains unchanged as it is symmetric when interchanging $\hat{\varepsilon }$ and $\hat{\mu }$ [31].

The diagonal elements of ${\hat{\varepsilon }}^{\prime }$ are then all equal to $n_{o}n_{e}$, corresponding to a material with a mixed refractive index $\sqrt{n_{o}n_{e}}$. However, the transformed equation for the *z* component of the magnetic field $\vec{H}$ reads

(14) $$\frac{\partial^{2}H_{z}}{\partial u^{2}}+\frac{\partial^{2}H_{z}}{\partial v^{2}}+\frac{\omega^{2}}{c^{2}}n_{e}^{2}H_{z}=0\;,$$

resembling the equation of an isotropic medium with the extraordinary refractive index $n_{e}$, in contrast to the entries of ${\hat{\varepsilon }}^{\prime }$. This can be understood by looking at the permeability tensor ${\hat{\mu }}^{\prime }$. For TE polarization, $\vec{H}$ equals $H_{z}$, and only the *z* component of ${\hat{\mu }}^{\prime }$ plays a role, $\mu_{zz}^{\prime }=\frac{1}{\beta }=\frac{n_{e}}{n_{o}}$. Multiplied by the isotropic ${\hat{\varepsilon }}^{\prime }$, it indeed yields a refractive index of $n_{e}$ in Equation (14).

Transforming the boundary of the disk accordingly (i.e., u=x,v=βy), we arrive at an ellipse

(15) $$u^{2}+\frac{n_{e}^{2}}{n_{o}^{2}}v^{2}=1\;,$$

with (unchanged) semi-axis lengths of one in the *x* (or *u*) direction, and a flattened semi-axis of $n_{o}^{2}/n_{e}^{2}\leq 1$ in the *y* (*v*) direction, to constitute the boundary of the transformed, isotropic cavity. We point out that the values of the semi-axes are the same as for the ellipse representing the dispersion relation in *k*-space, Equation (5), which was used for the ray simulation, as it should be. The preferred group velocities, perpendicular to the flat sides of the dispersion ellipse, correspond to the bouncing-ball orbits of the corresponding isotropic ellipse (representing the *transformed* disk), yielding a coherent view of the dynamics occurring in the anisotropic disk. It also provides some insight into the mathematical challenges when studying the anisotropic instead of the isotropic disk. The mathematical complexity is induced by going from a geometry with one center point (circle) for the isotropic disk to a (transformed) geometry with two focal points (ellipse). This can be seen as a bifurcation. Notice, however, that the limit is well defined when taking the opposite direction and going from an ellipse to an (isotropic) disc.

**Figure 6:**
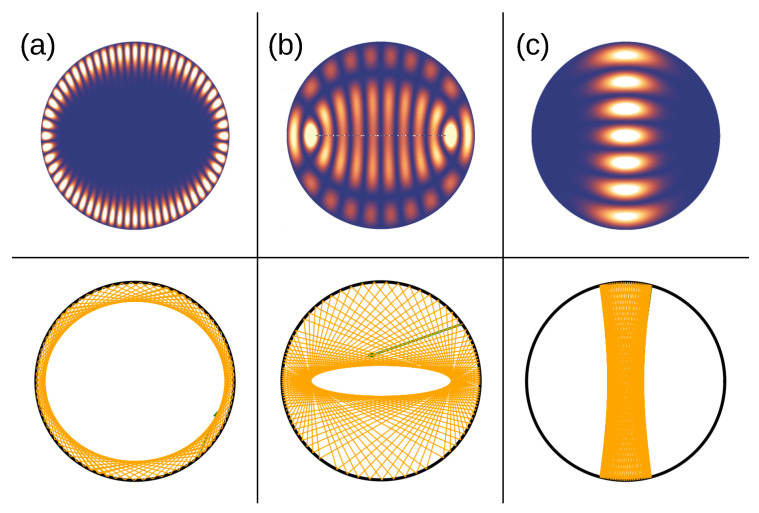
Ray–wave correspondence in the closed anisotropic disk. The upper (lower) row shows representative wave resonances (corresponding sample ray trajectories) for (**a**) a WG type resonance, (**b**) a resonance in the vicinity of the separatrix, and (**c**) a bouncing-ball mode.

**Figure 7:**
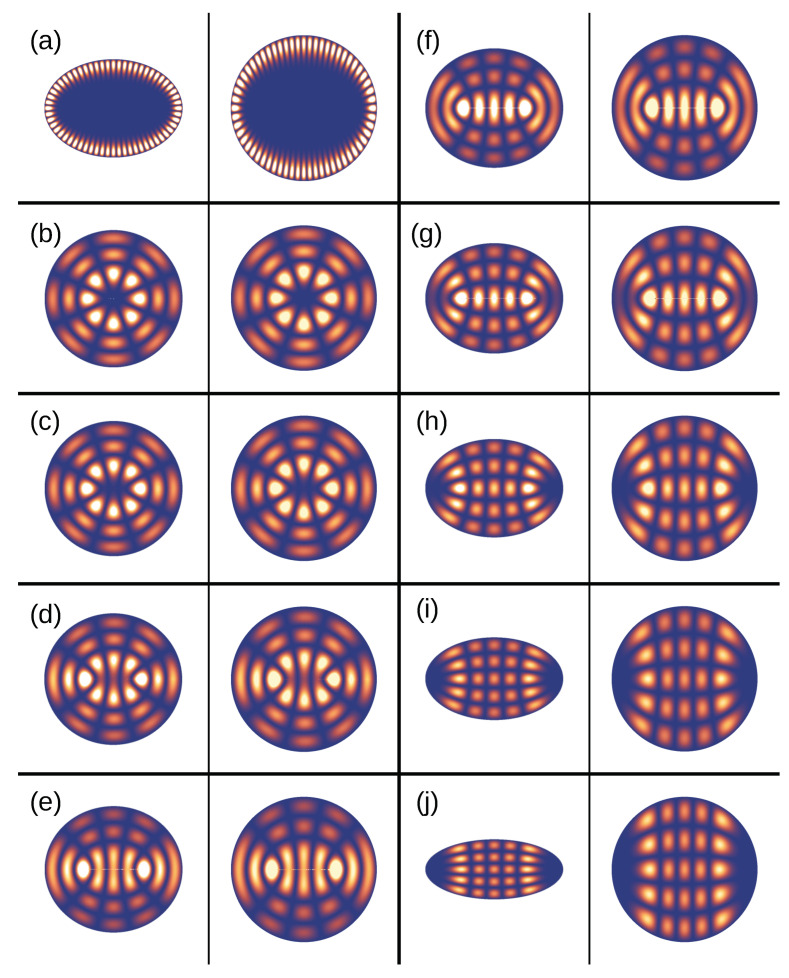
Resonance solutions of the isotropic ellipse (left panels) and the corresponding anisotropic disk (right panels) for different anisotropy strengths *a*, all for the closed system. Panel (**a**) shows a WG mode, illustrating the deviation of the wave pattern from the isotropic disk WG resonance case. Panels (**b**–**j**) show the evolution of a resonance with an angular momentum quantum number m=4 (corresponding to 2m=8 maxima) and a radial quantum number ρ=3 for increasing anisotropy: (**b**) a=0.1, (**c**) a=0.2, (**d**) a=0.3, (**e**) a=0.4, (**f**) a=0.5, (**g**) a=0.6, (**h**) a=0.7, (**i**) a=0.8, (**j**) a=0.9.

The solution of closed isotropic elliptical billiards is well known [37,38] and yields eigenmodes in the form of Mathieu functions (instead of Bessel functions, as for a closed isotropic disk), with quantum numbers *m* for the angular motion and ρ in the radial direction, and the requirement of continuity across the focal points of the ellipse. These solutions have to be mapped back to the original disk. By doing so, we arrive at the desired solutions for the anisotropic disk, which are shown in Figure 6 in qualitative comparison to their ray trajectory counterparts and in Figure 7 together with their isotropic ’elliptical’ counterparts. The more the (isotropic) ellipse geometry deviates from the circle, the more anisotropic is the disk cavity that it describes according to transformation optics.

## 4. Discussion: Ray–Particle-Wave Correspondence for Optical and Electronic Systems

What can we learn from the description of two different mesoscopic systems, an electronic and a photonic one? The issues are as follows. For the optical system, transformation optics relies on the invariance of Maxwell’s equations under coordinate transformations like the one we used here and has proven very useful in many cases. In contrast, a 4×4 Hamiltonian is needed to describe bilayer graphene; moreover, electrons are charged spinful particles. To highlight the differences that are of interest here, the particle tracing point of view is useful. In the PSOS in Figure 1(a.1,b.1), we had to distinguish two different surfaces of section, depending on the valley index of the BLG billiards ($K^{+}$ and $K^{-}$). It is straightforward to differentiate these cases when no intervalley scattering is present, as may be the case for low carrier densities and for external fields that lift the level of degeneracy. However, in general, both *K* valleys have to be taken into account. This is fulfilled in any tight-binding calculation [9] that is based on the full Hamiltonian of the system. Conversely, taking the transformation optics point of view and starting from a circular BLG billiard with a trigonally warped Fermi line, it is not guaranteed to find a non-circular BLG billiard geometry with a circular Fermi line that represents the same dynamics in a PSOS. In fact, it is impossible when only one valley ($K^{+}$ or $K^{-}$) is considered. The reason is that a PSOS corresponding to a triangularly warped Fermi line is *not* symmetric with respect to $k_{\|}=0$ [10,28], while it is symmetric for the associated isotropic but deformed billiard geometry. The consequence that changing the sense of rotation for an individual trajectory yields different dynamics in BLG billiards for a given pseudospin marks a distinct difference compared to the optical case.

This investigation of anisotropic BLG billiards provided the tools to study birefringent optical systems by extending ray optics to the anisotropic situation. This approach completes the wave/transformation optics point of view and establishes ray–wave correspondence for anisotropic billiards for light. To this end, we express the anisotropy of a uniaxial optical system in terms of the index ellipsoid as usual, and assume the *y* axis of a two-dimensional optical system in the (x,y) plane to be an optical axis with a refractive index $n_{e}>n_{o}$. We first used a ray-tracing approach motivated by BLG billiard studies and derived the dispersion relation (a quantity freely available in solid state physics)for the optical case, arriving at an ellipse in momentum space with the elongation in the $k_{x}$ direction. The latter becomes plausible when employing reciprocity arguments relating real and momentum space. In contrast, transformation optics does not require expedition to momentum space, though it arrives at the very same elliptical geometry now in real space in the case considered *here*. The reason is that we started with an anisotropic disk—its isotropic counterpart assigned by transformation optics is the isotropic elliptical cavity, which has the same shape as the dispersion relation. This occurs by chance in the sense that if we had started with a non-circular geometry, the transformation optics would yield a another transformed cavity shape, while the dispersion relation would remain unchanged. We note that in the case of BLG, changing the geometry of the cavity and the Fermi line has different effects on the overall system dynamics [39].

For non-circular cavities, the ray–particle tracing approach proves to be particularly useful as it treats any given anisotropy that can be combined with an arbitrary billiard geometry using the very same algorithm, and ray–wave correspondence is applied to generalize the results to describe the wave counterpart. When working on the wave side, transformation optics is ideally suited to transform anisotropic *wave* problems to isotropic ones that are much easier to solve. The knowledge gained from the (numerically cheap) tracing simulations can provide useful guidance in the interpretation of results.

We focused on uniaxial and closed optical systems; in particular, we assumed the optical axis to lie in the resonator plane, representing the most interesting yet simple case. Generalization to the generic case of an arbitrary pointing optical axis that does not coincide with the resonator plane, as illustrated in Figure 2a, is conceptually straightforward though tedious [29]. Here, we limited our consideration to the case of closed mesoscopic cavities. The important topic of realistic open systems will be discussed elsewhere.

## Data Availability

The data that support the findings of this study are available upon reasonable request from the authors.
